# Investigation of Genetic Causes of Intellectual Disability in Kerman Province, South East of Iran

**Published:** 2012-02-01

**Authors:** M J Soltani Banavandi, K Kahrizi, F Behjati, M Mohseni, H Darvish, I Bahman, S S Abedinni, S Ghasemi Firouzabadi, E Jafari, Sh Ghadami, F Sabbagh, Gh R Kavoosi, H Najmabadi

**Affiliations:** 1 Faculty of Basic Science, Science and Research Branch, Islamic Azad University, Fars, Iran; 2 Genetics Research Center, University of Social Welfare and Rehabilitation Sciences, Tehran, Iran; 3 Deptartment of Microbiology, Faculty of Basic Science, Islamic Azad University, Kerman Branch, Kerman, Iran; 4 Genetics Counseling Center, Welfare Organization of Kerman Province, Kerman, Iran; 5 Institute of Biotechnology, University of Shiraz, Shiraz, Iran

**Keywords:** Intellectual disability, Microcephaly, Iran

## Abstract

**Background:**

Intellectual disability (ID) has a worldwide prevalence of 1-3% and results from extraordinary heterogeneous. To shed more light on the causes of ID in Kerman Province, in Southeast Iran, we set out in 2008 to perform systematic clinical studies and homozygosity mapping in large Iranian families with ID.

**Methods:**

Fifty seven families with a minimum of two mentally retarded children from Kerman Province were initially tested for metabolic disorders, by Tandem mass spectrometry. Fragile X testing and standard karyotyping were performed for all probands of families. Cases with autosomal recessive (AR) pattern of inheritance and microcephaly were subjected to homozygosity mapping by using several microsatellite markers for known MCPH loci.

**Results:**

Three out of seven families with X-linked pattern of inheritance were positive for fragile X syndrome. Chromosome abnormality was not observed in any of dysmorphic patients and all families were negative for metabolic tests. Among the remaining 50 families of AR ID, six were found to be microcephalic, of which 2 linked to two MCPH loci (33.3%). The rest 4 families were not linked to any of the known loci.

**Conclusion:**

The results of this study showed that ID with microcephaly comprised 12% of ID cases in Kerman Province. In two families with apparent linkage to the MCPH5 and MCPH6 locus, mutation screening was not successful, which might indicate that either the mutation is located in the regulatory sequences of the gene or that there might be another genes present in these regions, which is mutated in such cases.

## Introduction

Defining feature of Intellectual disability (ID) includes Intelligence Quotient (IQ) below 70, significant limitation in two or more adaptive skill areas, and condition present from childhood.[1] ID is divided into two types: syndromic form which in addition to ID, other clinical features such as skeletal abnormality, facial dysmorphic features, etc. are seen, and nonsyndromic form that it is just accompanied with ID as clinical feature of disorder.[2] About two-thirds of all kinds of ID are due to genetic causes that include monogenic disorder and chromosomal abnormality. Chromosomal aberrations are one of the most important causes of ID. Numerical and structural abnormalities are responsible for about 4-28% of all ID patients,[3] and are found in about 40% of severe ID and 10% of mild ID cases.[4] In addition to the severity of ID, the presence of congenital anomalies increases the diagnostic yield of chromosome abnormalities.[3] ID is also divided into two mode of inheritance pattern, involving X-linked and autosomal.[5] X-linked Intellectual Disability (XL ID) which is estimated to be responsible for 5-12% of ID patients, for example fragile-X syndrome is the most common form of inherited ID after Down syndrome. Up to now, more than 80 known and candidate genes were discovered to be related to syndromic and non syndromic XL ID.[6][7][8]

Autosomal recessive intellectual disability (AR ID) has not been studied extensively basically because of lack of large consanguineous families with enough affected individuals for linkage analysis in western population. The number of known genes is still so limited[9][10][11] and up to now, just 23 loci and 13 genes have been found for non-syndromic AR ID.[12][13][14] As described earlier,[15] homozygosity mapping in 78 consanguineous Iranian families with non-syndromic autosomal recessive intellectual disability (NS-AR ID) determined the chromosomal localization of at least 8 novel gene loci for this condition, suggesting that AR ID is very heterogeneous. Recently, we identified 27 single linkage intervals, at least 14 novel loci and several mutation hotspots in Iranian AR ID patients by homozygosity mapping.[16] AR ID associated with primary microcephaly (MCPH) accounts for 15% of all IDs.[17] So far, seven loci have been found which associated with autosomal recessive primary microcephaly (MCPH1-MCPH7) and only five genes for them have been identified including Microcephalin at MCPH1, CDK5RAP2 at MCPH3, ASPM at MCPH5, CENPJ at MCPH6, and STIL/SIL at MCPH7.[18][19][20] So far, several mutations have been identified for these loci in different countries as well as Iran[17][21] but, as we faced an utter lack of data in this area in Kerman Province, with a high consanguinity marriage and mentally retarded families, we decided to investigate the genetic causes of the disease in this part of the country.

## Materials and Methods

Our study included a total of 57 families comprising intellectually disabled patients with majority of them having two or more affected individuals in their families. Each family received a complete clinical examination and environmental causes of microcephaly were excluded. The parents had normal head circumference and intelligence and microcephaly in the affected children was present at birth. DNA was extracted from peripheral blood samples following a standard protocol.[22] Cytogenetics analysis was performed on cultured peripheral blood lymphocytes stimulated with phytohemagglutinin M, using standard techniques.[23] The karyotype was determined in dysmorphic or microcephalic patients by GTG banding technique. Informed consent was obtained for research, following the procedures approved by the institute’s ethical committee.

Fragile X testing was performed using PCR and/or southern blot analysis.[24] To exclude chromosomal abnormalities and metabolic disorders, respectively, we performed standard G-band karyotyping and tandem mass spectrometry, from at least one patient of each nuclear family.[25][26] A panel of 31 microsatellite markers was selected from the Genome Database (http://www.gdb.org/) and Marshfield Medical Research Foundation (http://www.research.marshfieldclinic.org/genetics/).

Due to differences in allelic frequency of markers in every population, 10 individuals of different ethnic groups were studied for allelic heterozygosity. Polyacrylamide gel electrophoresis and a standard silver stain protocol were used to visualize the results. When the haplotype at a given MCPH locus was found to be homozygous in all affected individuals of a family mutation screening was initiated. If heterozygous markers or different homozygous haplotypes were found in the patients, the respective locus was excluded. The probands of the family linked to the MCPH5 locus on chromosome 1q31, all 28 exons and exon/intron splice junctions of the ASPM gene (National Center for Biotechnology Information GenBank Accession Number AF509326) were sequenced, using a set of 33 PCR primers.[21] After PCR amplification, the amplicons[23] were sequenced using an ABI 3730 genetic analyzer (Applied Biosystems, Foster city, California, USA.). Sequences were compared with the reference genomic and the reference cDNA sequence (NM_018136). For the other family linked to MCPH6 locus on chromosome 13q12.2, using a set of 17 primers (21), designed with the Primer 3 software,[27] the 17 exons and exon/intron splice junctions of the CENPJ gene (GenBank accession number BC024209) were amplified and sequenced as described previously.[21]

## Results

As the first investigation on genetic causes of ID in Kerman Province of Iran, a total of 57 families with two and/or more affected individuals including 157 ID patients from different parts of the province have been collected. Sex distribution and clinical findings among the studied population were shown in Table 1. Thirty seven out of 57 families had non-syndromic phenotype and 4 syndromic; of whom three families were Fragile X syndrome and one Bardet-Biedel syndrome. Apart from ID, 16 families showed consistent additional features such as microcephaly, sensory neural hearing loss, neurologic symptoms including seizure, ataxia, spasticity, blindness and increased deep tendon reflexes. Mass spectrometry did not show abnormalities in any of the investigated patients.

**Table 1:** Demographic and clinical data derived from 57 studied ID families of Kerman Province

**State**	**Number of families**	**Percentage**
Marriage		
Consanguineous	38	65.5
Non-consanguineous	20	34.5
Pattern of inheritance		
Autosomal recessive	51	87.93
X-linked	7	12.07
Other clinical features		
Syndromic	4	6.9
Nonsyndromic	37	63.8
ID+additional features	17	29.3
Number of affected individuals		
2 affected	26	32.5
>2 affected	32	67.5
Sex		
Male	68	42.5
Female	92	57.5

**Fig. 1:**
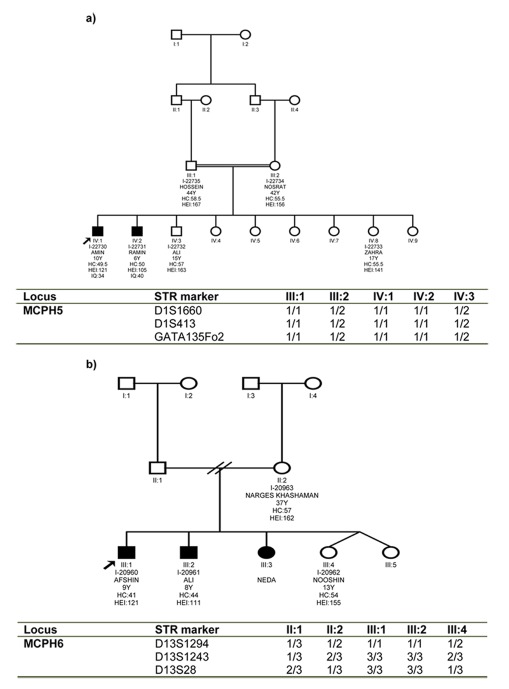
Pedigree, haplotype analysis of the core families linked to MCPH5 (a), and MCPH6 (b).

Homozygosity mapping for seven MCPH loci in 6 out of 57 families (10.5%) with patients suffering from primary microcephaly led to the identification of three families each linked to one MCPH loci; MCPH5, and MCPH6. The pedigree and haplotype analysis of families were shown in Figure 1. The degree of ID in the affected family members ranged from severe to profound (Table 2). None of the patients showed any other neurological problems, congenital malformations, or facial dysmorphisms. All patients in two families showed <3 SD head circumference. The affected individuals in M-8700149 and M-8700061 suffered moderate and severe ID, respectively. No linkage pattern to MCPH loci was observed in the rest families.

**Table 2:** Clinical features of the two ID families linked to MCPH5 and MCPH6 of Microcephaly loci.

**Patient **	**Sex**	**Age at examination (yrs)**	**Mental retardation (IQ)**	**Height (Cm)**	**OFC^a^ (Cm)**	**Other features**
MCPH5						
IV :1	Male	10	Sever (34)	121	-3SD	------------
IV :2	Male	6	Sever (40)	125	-3SD	------------
MCPH6						
III :1	Male	9	Moderate	121	-3SD	------------
III :2	Male	8	Moderate	111	-3SD	------------

^a^ OFC denotes occipitofrontal circumference.

## Discussion

Three years ago, we have joined forces to elucidate the molecular causes of ID in Kerman Province, Southeastern Iran. In this population, almost 40% of all children are born from consanguineous parents, and family sizes are much larger than in Western societies. As our first objective, we have tried to identify, or rule out, common forms of AR ID by performing large-scale homozygosity mapping.

A total of 57 Iranian families with 157 mentally retarded patients have been investigated. High-resolution G-banding chromosomal analysis demonstrated a normal karyotype in all patients. According to the results of previous studies in our center, the prevalence of chromosome abnormality in Iranian ID patients ranging from 1.24% to 7% depending on selection criteria (unpublished data). The former being only in consanguineous families with idiopathic ID. In countries with a high proportion of consanguineous marriages such as Iran (about 65% in this study), it is more likely that ID to be as a result of genetic heterogeneity of autosomal recessive forms of ID than chromosomal abnormality. Mass spectrometry did not show abnormalities in any of the investigated patients, which is in keeping with earlier reports that in familial ID, errors of metabolism are rare.[15][28][29]

Among 57 ID families, three were identified as fragile X syndrome. Because in males, ID is significantly more frequent than in females, it had been assumed that defects of X-linked genes account for up to 25% of severe cases, but recent data suggest that the contribution of X-linked defects is much lower, probably in the range of 10%.[30] The frequency of 5.2% in this study is comparable to other reports from Iran and neighboring countries such as India.[31][32][33]

A variety of neurologic symptoms in nine families have been detected, one family showed ID plus sensory neural hearing loss in all affected members, one family with Bardet-Biedle syndrome and six out of 57 families had ID associated with microcephaly (12%). Microcephaly is a clinical finding that defined as a reduction in head circumference which refers to decrease of brain volume in affected individuals. Microcephalic patients have head circumferences below -3 standard deviation (SD) and most of them are mentally retarded,[17] but with no other neurological findings such as spasticity or progressive cognitive decline. Height, weight, appearance, chromosomal analysis and brain scan are normal, but do not exclude the diagnosis.

This is the first study of the genetics of primary microcephaly in a cohort of families in Kerman Province of Iran. Linkage analysis revealed that 2 out of 7 microcephal families (33.3%) were linked to two MCPH loci; MCPH5, and MCPH6. ASPM mutations (MCPH5) were the most common known cause for primary microcephaly followed by mutations at the MCPH2 and MCPH1 locus.[21] As recently presented, we were able to find one missense mutation (c.215C>T/ p.S72L) in exon 3 of Microcephalin gene (MCPH1) located in the BRCT1 domain of the microcephalin protein in a family originated to Kerman Province with three microcephalic patients.[21] In two families with apparent linkage to the MCPH5 and MCPH6 locus, no mutations in ASPM and CENPJ genes were detected, which might indicate that either the mutation is located in regulatory sequences of the gene, or that there might exist another microcephaly gene in this region. Further sequence analysis of promoter, 3'UTR and 5'UTR regions in these two genes is underway. Recently, it has been shown that MCPH5, MCPH1, and MCPH6 were the most prevalent MCPH loci in Iran with a frequency of 13.3%, 8.2%, and 5.1%, respectively.[21] They were also the most common loci in our neighboring countries; MCPH5 is the most frequent loci among microcephal populations in Pakistan (accounts for 43% to 86% of loci), and India (33.5%).[27][34][35]

Our results indicate that ID with microcephaly comprises about 12% of ID cases in Kerman Province. Fragile X syndrome was the second most prevalent finding in our ID families. Overall, we could explain the genetic causes of ID in ~7% of our families and the remaining need to be further investigated.
